# Effectiveness and safety of awake prone positioning in COVID-19-related acute hypoxaemic respiratory failure: an overview of systematic reviews

**DOI:** 10.1186/s12890-023-02829-2

**Published:** 2024-01-02

**Authors:** Ya Li, Guixiang Zhao, Yizhao Ma, Lu Wang, Ying Liu, Hailong Zhang

**Affiliations:** 1 Co-Construction Collaborative Innovation Center for Chinese Medicine and Respiratory Diseases by Henan & Education Ministry of P.R. China, Henan University of Chinese Medicine, Zhengzhou, 450046 China; 2 Henan Key Laboratory of Chinese Medicine for Respiratory Disease, Henan University of Chinese Medicine, Zhengzhou, 450046 China; 3 Department of Respiratory Diseases, The First Affiliated Hospital of Henan University of Chinese Medicine, Zhengzhou, China

**Keywords:** COVID-19, Acute hypoxemic respiratory failure, Awake prone positioning

## Abstract

**Objective:**

To evaluate and summarize systematic reviews of the effects and safety of awake prone positioning for COVID-19-related acute hypoxaemic respiratory failure.

**Methods:**

A comprehensive search was conducted on PubMed, Embase, the Cochrane Library, Web of Science, CNKI, CSPD, CCD and CBM from their inception to March 28, 2023. Systematic reviews (SRs) of awake prone positioning (APP) for COVID-19-related acute hypoxaemic respiratory failure in adults were included. Two reviewers screened the eligible articles, and four reviewers in pairs extracted data and assessed the methodological quality/certainty of the evidence of all included SRs by AMSTAR 2 and GRADE tools. The overlap of primary studies was measured by calculating corrected covered areas. Data from the included reviews were synthesized with a narrative description.

**Results:**

A total of 11 SRs were included. The methodological quality of SRs included 1 “High”, 4 “Moderate”, 2 “Low” and 4 “Critically low” by AMSTAR 2. With the GRADE system, no high-quality evidence was found, and only 14 outcomes provided moderate-quality evidence. Data synthesis of the included SR outcomes showed that APP reduced the risk of requiring intubation (11 SRs) and improving oxygenation (3 SRs), whereas reduced significant mortality was not found in RCT-based SRs. No significant difference was observed in the incidence of adverse events between groups (8 SRs). The corrected covered area index was 27%, which shows very high overlap among studies.

**Conclusion:**

The available SRs suggest that APP has benefits in terms of reducing intubation rates and improving oxygenation for COVID-19-related acute hypoxemic respiratory failure, without an increased risk of adverse events. The conclusion should be treated with caution because of the generally low quality of methodology and evidence.

**Trial registration:**

The protocol for this review was registered with PROSPERO: CRD42023400986. Registered 15 April 2023.

**Supplementary Information:**

The online version contains supplementary material available at 10.1186/s12890-023-02829-2.

## Background

The global COVID-19 pandemic, caused by severe acute respiratory syndrome coronavirus-2, has resulted in devastating medical, economic, and social consequences. According to the World Health Organization (WHO), as of January 2023, COVID-19 has impacted approximately 672 million individuals and caused 6.7 million deaths globally (Coronavirus COVID-19 (2019-nCoV) (arcgis.com). Acute respiratory distress syndrome (ARDS) is characterized by severe respiratory distress and refractory hypoxemia, which is a contributing factor to both mechanical ventilation requirements and mortality among COVID-19 patients [1, 2]. Research studies have reported an in-hospital mortality ranging from 34.9 to 46.1% [3] among ARDS patients, with the case-fatality rate reaching approximately 50% [4] for COVID-19 patients with ARDS.

Prone positioning (PP), which involves placing the patient in a prone posture, has been confirmed as an effective treatment approach for ARDS patients [5, 6]. Its mechanism involves enhancing the even distribution of gas throughout the lungs, optimizing the ventilation/perfusion ratio, facilitating re-expansion of collapsed dorsal alveoli, and preventing excessive inflation of normal alveoli. This approach effectively ameliorates hypoxemia, corrects hypercapnia, and significantly enhances survival outcomes [6–9]. The utilization of APP has been extensively employed in patients with acute hypoxemic respiratory failure related to COVID-19 since the emergence of the pandemic [10, 11]. International guidelines recommend APP as a standard treatment for suspected or confirmed COVID-19 patients due to its potential clinical benefits [12–14]. Several SRs have been published to evaluate the effect of APP on clinical outcomes in COVID-19-associated acute hypoxemic respiratory failure. However, discrepancies in the conclusions drawn from various SRs exist, highlighting the need for a thorough evaluation of their quality. This study aims to provide a comprehensive overview of the methods and evidence quality of SRs on COVID-19-related acute hypoxemic respiratory failure, with the purpose of offering valuable references for clinical practice.

## Methods

The present study was carried out in accordance with the Cochrane guidelines for overview of reviews [15] and we adhered to the Preferred Reporting Items for Overviews of Reviews – PRIOR checklist (Appendix S1) [16, 17]. The protocol for this review was registered with PROSPERO: CRD42023400986. Registered 15 April 2023.

### Inclusion criteria

#### Study design

Systematic Review and Meta-analysis Based on Clinical Studies.

#### Study population

The study enrolled adult patients diagnosed with COVID-19-associated acute hypoxemic respiratory failure, without any gender, age, disease duration, case source, country of origin or ethnicity restrictions.

#### Interventions

The intervention group in this study received treatment with awake prone positioning (APP), with or without additional therapies such as oxygen therapy, and other relevant interventions, while the control group received non-APP treatment.

#### Outcome measures

Intubation risk, all-cause mortality, oxygenation, ICU length of stay, hospital length of stay, ventilator-free day, safety outcomes.

### Exclusion criteria

Non-Chinese and non-English publications, duplicate or redundant data from the same study, conference abstracts lacking corresponding full-text articles, and systematic reviews that are still in the planning or title stage without published results will be excluded.

### Search strategy

Two investigators (YL and GXZ) searched four English databases (PubMed, Embase, the Cochrane library, Web of Science) and four Chinese databases (CNKI, CSPD, CCD, CBM) from their inception to March 28, 2023. The search strategies were designed based on subjective terms and free terms for each topic and were adapted for each database when conducting the search. Detailed retrieval strategies and steps are presented in Appendix S2.

### Data extraction and synthesis

The screening process for titles, abstracts, and full texts was conducted independently by two investigators (YL and GXZ). Any discrepancies in screening or extraction were resolved through consensus with a third author. Data extraction involved utilizing an Excel data sheet that had been predesigned: 1. Basic information: Author, year of publication, nationality, number of original studies included, sample size, interventions, quality assessment tools, outcomes, etc. 2. Methodological quality of the SRs: Relevant information regarding the methodological quality of the systematic reviews was extracted. 3. Statistical analysis results: The qualitative or quantitative analysis results of each outcome measure were the primary focus of data extraction. 4. It is critically difficult to conduct a meta-analysis because of high heterogeneity in the population, intervention, study designs and outcomes, among the included SRs, Therefore, we summarized the data from the individual reviews narratively and presented these summaries using tables.

### Calculation of the CCA for overlapping area

The corrected covered area (CCA) was calculated to provide a measure of the extent to which primary studies overlap in the included SRs [18]. The following calculation was used: CCA = N − r/rc − r. N indicates the number of included publications, r indicates the number of included publications, and c is the total number of SRs. The final value was then converted to a percentage of overlap.

### Quality assessment

#### Quality appraisal

The methodological quality of the included systematic reviews was assessed using the AMSTAR-2 tool [19], which consists of 16 items, with items 2, 4, 7, 9, 11, 13, and 15 considered critical items. Each item is evaluated as “Yes” (indicating that the criterion is met), “No” (indicating that the criterion is not met), or “Partial Yes” (indicating that the criterion is partially met). Based on the evaluation results of both critical and noncritical items, the methodological quality of the systematic review could be categorized into four levels: high, moderate, low, or critically low.

#### Evaluation of evidence quality

The GRADE system was used to evaluate the quality of evidence, classifying a study into one of four levels: high, moderate, low, or very low. The GRADE system initially classifies randomized controlled trials as “high” quality evidence and observational studies as “low” quality evidence. The grade was assessed based on five factors, including limitations, inconsistency, indirection, imprecision and publication bias of the study. Alternatively, it could be evaluated based on two factors: large effect and consistency of the study results. Two researchers independently assessed the evidence quality. Any disagreements were resolved through discussion with a third researcher.

## Results

### Literature screening process and results

**Fig. 1:**
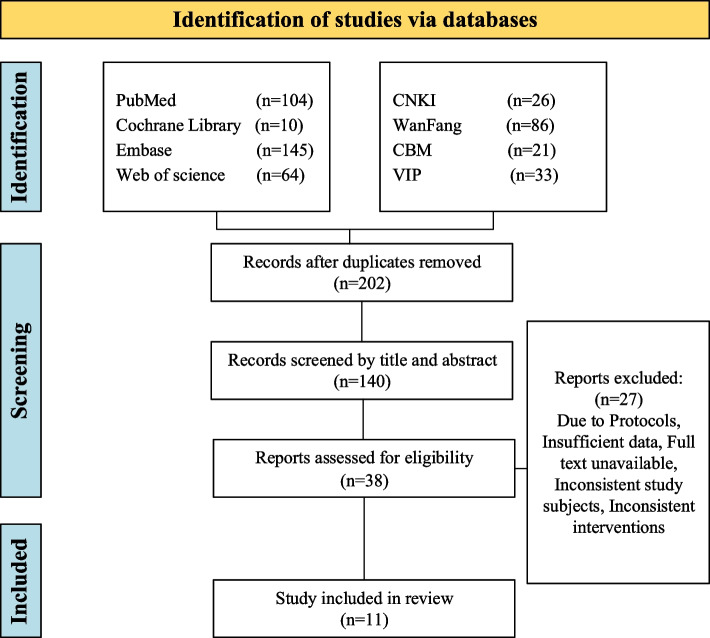
Study selection flowchart

The literature search initially retrieved 489 articles. After removing duplicates, 287 articles were excluded. Following the screening of titles and abstracts, 62 articles were excluded. After full-text review, 102 articles were further excluded. Finally, a total of 11 articles [20–30] were included in the analysis. The flowchart outlining the search process is presented in Fig. 1.

### The basic characteristics of the included studies

The review included 11 studies [20–30] published between 2022 and 2023, with the number of primary studies ranging from 8 to 35 and sample sizes varying from 1401 to 6311 participants. Six studies [20, 21, 27–30] exclusively comprised RCTs, while the other five studies consisted of a combination of RCTs and observational studies. Various forms of initial respiratory support were utilized in the included studies. Only 1 meta-analysis [23] exclusively enrolled patients from ICU settings, while the remaining studies recruited patients from wards, emergency departments (EDs), units, or other locations. The median duration of prone positioning in the included studies within the APP group ranged from 1 hour to 12 hours per day. Methodological quality assessment of the included studies showed that 10 SRs [20–24, 26–30] employed the Cochrane Collaboration risk of bias tool for RCTs, one study [25] utilized the Jadad composite scale to evaluate the methodological quality of RCTs, and four studies [22–25] used the Newcastle–Ottawa Scale to assess the risk of bias in observational studies. Among all the included studies, 11 studies [20–30] evaluated intubation rates and all-cause mortality as outcome measures, 3 studies [20, 23, 30] focused on improvement in oxygenation parameters, 5 studies [21, 22, 26, 27, 29] examined ICU length of stay, 7 studies [21, 22, 25–27, 29, 30] investigated hospital length of stay, 3 studies [21, 26, 27] measured ventilator-free days, and 8 studies [21, 22, 24, 26–30] reported adverse events. Details of the characteristics of the included SRs are shown in Table 1. Summary of Subgroup Analysis Results reported by the reviews is presented in Table 2.

**Table 1:** Basic features to be included in systematic reviews

ID	Population	No. of included studies (ss)	Study design	Enrolment location	Intervention	Control	Risk of bias evaluation tool	Outcomes
Santa Cruz 2022 [20]	Non-Intubated	8(1401)	RCT	ICU, medical ward	APP	usual care	Cochrane Risk of Bias tool	①②③
Cheema 2023 [21]	Non-Intubated	11(2385)	RCT	ICU, medical ward	APP	supine position	Cochrane Risk of Bias tool	①②④⑤⑥⑦
Li 2022 [22]	Non-Intubated	10(1985) 19(2669)	RCTs, observational studies	ICU, medical ward, emergency department,	APP	supine position	Cochrane+NOS	①②④⑤⑦
Huang 2022 [23]	Non-Intubated	10(1686) 12(1522)	RCT, observational studies	ICU	APP	supine position	Cochrane+NOS	①②③④
Kang 2022 [24]	Non-Intubated	7(2364) 15(2782)	RCTs, observational studies	ICU or ED or Ward or monitored acute care unit.	APP	supine position	Cochrane + NOS	①②⑦
Beran 2022 [25]	Non-Intubated	14(3324)	RCT, retrospective cohort, prospective cohort	ICU, ward, ED	APP	supine position	NOS + Jadad composite scale	①②⑤
Lee 2022 [26]	–	9(2431) 23(3880)	RCT, prospective cohort studies, retrospective cohort studies	unit, ICU, Ward ER	prone position	non-prone position	Cochrane	①②④⑤⑥⑦
Weatherald 2022 [27]	Non-Intubated	17(2931)	RCT	Medical ward, ICU, HDU	APP	usual care	Cochrane	①②④⑤⑥⑦
Wang 2023 [28]	Non-Intubated	10(2294)	RCT	ward or ICU	APP	usual care	Cochrane	①②⑦
Cao 2023 [29]	–	8(2657)	RCT	ward or ICU	APP for at least 6 h a day	usual care	Cochrane	①②④⑤⑦
Peng 2023 [30]	–	13(3263))	RCT	ward or ICU	APP	usual care	Cochrane	①②③⑤⑦

*ED* emergency department, *HDU* high dependency unit, *RCT* randomised controlled trial, *ICU* intensive care unit. ①Intubation Rate ②Mortality ③Oxygenation ④ICU Length of Stay ⑤Hospital Length of Stay ⑥Ventilator-Free Days (VFD) ⑦Adverse Events

**Table 2:** Summary of subgroup analysis results

Study	Outcome	Subgroup		Numbers	MD(RR/OR)	Heterogeneity	*p* value
Cheema 2023 [21]	Intubation rate	type of respiratory support	higher level of respiratory support	4 RCT(765/756)	RR 0.82[0.71, 0.93]	0%	0.29
			conventional oxygen therapy	9 RCT(450/411)	RR 1.07[0.66, 1.73]	0%	
		enrollment location	non-ICU	7 RCT(394/355)	RR 0.88[0.44, 1.76]	0%	0.87
			ICU	4 RCT(788/773)	RR 0.83[0.73, 0.95]	0%	
	Mortality	type of respiratory support	higher level of respiratory support	4 RCT(810/799)	RR 0.92[0.76,1.10]	0%	0.64
			conventional oxygen therapy	8 RCT(405/368)	RR 1.14[0.47,2.75]	0%	
		enrollment location	ICU	4 RCT(808/773)	RR 0.91[0.75,1.10]	0%	0.75
			non-ICU	7 RCT(394/355)	RR 0.81[0.41,1.59]	0%	
Li 2022 [22]	Intubation rate	type of respiratory support	higher level of respiratory support	3 RCT(605/604)	RR 0.83[0.71, 0.97]	0%	0.88
			conventional oxygen therapy	8 RCT(405/368)	RR 0.87 [0.45, 1.69]	0%	
		enrollment location	ICU	3 RCT(583/578)	RR 0.83 [0.71, 0.97]	0%	0.86
			non-ICU	7 RCT(394/355)	RR 0.88 [0.44, 1.76]	0%	
	Mortality	type of respiratory support	higher level of respiratory support	3 RCT(605/604)	RR 1.23 [0.54, 2.80]	32%	0.90
			conventional oxygen therapy	8 RCT(405/368)	RR 1.14 [0.47, 2.75]	0%	
		enrollment location	ICU	3RCT(583/578)	RR 0.90 [0.72, 1.13]	0%	0.77
			non-ICU	7 RCT(394/355)	RR 0.81 [0.41, 1.59]	0%	
	ICU length of stay	type of respiratory support	higher level of respiratory support	3 RCT(401/441)	MD −0.53[−1.82, 0.75]	0%	–
			conventional oxygen therapy	3 RCT(68/67)	MD 0.76[−0.62, 2.13]	0%	–
		enrollment location	ICU	3 RCT(583/578)	MD 0.34[−0.77, 1.45]	0%	–
			non-ICU	2 RCT(57/54)	MD −0.99[−2.69, 0.71]	0%	–
	Hospital length of stay	type of respiratory support	higher level of respiratory support	3 RCT(605/604)	MD −0.35[−1.53, 0.83]	39%	–
			conventional oxygen therapy	6 RCT(252/216)	MD 1.15[0.26, 2.05]	0%	–
		enrollment location	ICU	2 RCT(553/548)	MD 0.22[−1.55, 2.00]	26%	–
			non-ICU	6 RCT(268/233)	MD 1.16[0.27, 2.05]	0%	–
Kang 2022 [24]	Intubation rate	type of respiratory support	conventional oxygen therapy	4 RCT(51/77)	OR 1.04[0.22, 4.87]	0%	0.51
			HFNC/NIV	5 RCT(1058/1102)	OR 0.60[0.39, 0.93]		
		daily median duration	>8H	5 RCT(519/568)	OR 0.47[0.25, 0.88]	65.5%	0.09
			<8H	8 RCT(1277/1264)	OR 0.85[0.65, 1.12]		
	Mortality	type of respiratory support	conventional oxygen therapy	4 RCT(120/185)	OR 0.37[0.17, 0.81]	55.1%	0.14
			HFNC/NIV	5 RCT(1052/1080)	OR 0.76[0.46, 1.26]		
		daily median duration	>8H	5 RCT(513/546)	OR 0.65[0.31, 1.34]	0%	0.49
			<8H	7 RCT(1231/1230)	OR 0.85[0.65, 1.11]		
Lee 2022 [26]	Mortality	type of respiratory support	Nasal cannula or facial mask	3 RCT(183/165)	RR 1.13[0.31, 5.70]	0%	0.61
			HFNC/NIV	5 RCT(1036/1020)	RR 0.91[0.78, 1.05]		
		type of respiratory support	Nasal cannula or facial mask	6 non-randomized studies(700/609)	RR 0.57[0.48, 0.67]	0%	0.40
			HFNC/NIV	6 non-randomized studies(405/857)	RR 0.47[0.31, 0.71]		
	Intubation rate	type of respiratory support	Nasal cannula or facial mask	2 RCT(57/43)	RR 1.00[0.28, 3.63]	0%	0.74
			HFNC/NIV	5 RCT(1036/1020)	RR 0.80[0.72, 0.90]		
		type of respiratory support	Nasal cannula or facial mask	5 non-randomized studies(640/506)	RR 0.74[0.41, 1.33]	0%	0.53
			HFNC/NIV	6 non-randomized studies(411/879)	RR 0.60[0.42, 0.85]		
Weatherald 2022 [27]	Intubation rate	daily median duration	≥5 h	3 RCT(457/448)	RR 0.78[0.66, 0.93]	0%	0.72
			<5 h	7 RCT(489/480)	RR 0.92[0.76, 1.12]		
		median baseline oxygen saturation to fraction of inspired oxygen (SpO_2_:FiO_2_)	SpO_2_:FiO_2_ < 150	2 RCT(421/409)	RR 0.77[0.64, 0.92]	0%	0.85
			SpO_2_:FiO_2_ ≥ 150	10 RCT(1151/1107)	RR 0.92[0.77, 1.10]		
		type of respiratory support	high flow or NIV	9 RCT(805/778)	RR 0.81[0.71, 0.92]	0%	0.74
			mixed	3 RCT(187/182)	RR 1.07[0.49, 2.34]		
			low flow	3 RCT(219/192)	RR 1.18[0.63, 2.19]		
		enrollment location	location mixed	6 RCT(588/576)	RR 0.81[0.69, 0.95]	0%	0.83
			ICU	4 RCT(292/275)	RR 0.86[0.69, 1.08]		
			in ward	4 RCT(331/301)	RR 0.96[0.43, 2.13]		
		Economic Co-operation and Development in 2021	low or middle income countries	3 RCT(291/274)	RR 0.69[0.55, 0.87]	0%	0.83
			High income countries	11 RCT(920/878)	RR 0.89[0.77, 1.04]		
Wang 2022	Intubation rate	SpO_2_/FiO_2_ ratio at baseline	SpO_2_/FiO_2_ > 235 mmHg	4 RCT(310/288)	RR 0.93[0.40, 2.19]	0%	–
			SpO_2_/FiO_2_ ≤ 235 mmHg	4 RCT(1021/1005)	RR 0.80[0.71, 0.90]	0%	–
	Mortality	SpO_2_/FiO_2_ ratio at baseline	SpO_2_/FiO_2_ > 235 mmHg	4 RCT(214/196)	RR 1.32[0.44, 2.99]	0%	–
			SpO_2_/FiO_2_ ≤ 235 mmHg	4 RCT(1021/1005)	RR 0.91[0.78, 1.06]	0%	–
Cao 2023 [29]	Intubation rate	Oxygen supply	HFNC	4 RCT(1021/1005)	OR 0.69[0.58, 0.83]	0%	–
	Mortality	Oxygen supply	HFNC	4 RCT(1144/1100)	OR 0.86[0.79, 1.05]	0%	–
Peng 2023 [30]	Intubation rate	daily median duration	>8H	9 RCT(1218/1172)	OR 0.76[0.63, 0.91]	0%	0.18
			<8H	4 RCT(456/417)	OR 0.59[0.42, 0.82]	0%	
		enrollment location	ICU	6 RCT(1064/1022)	OR 0.73[0.61, 0.88]	0%	0.61
			non-ICU	7 RCT(610/567)	OR 0.72[0.61, 0.84]	0%	
		type of respiratory support	conventional oxygen therapy	9 RCT(450/411)	OR 1.05[0.59, 1.86]	0%	0.12
			HFNC/NIV	6 RCT(1026/1000)	OR 0.65[0.54, 0.78]	0%	

### Replication rate of the original study

**Fig. 2:**
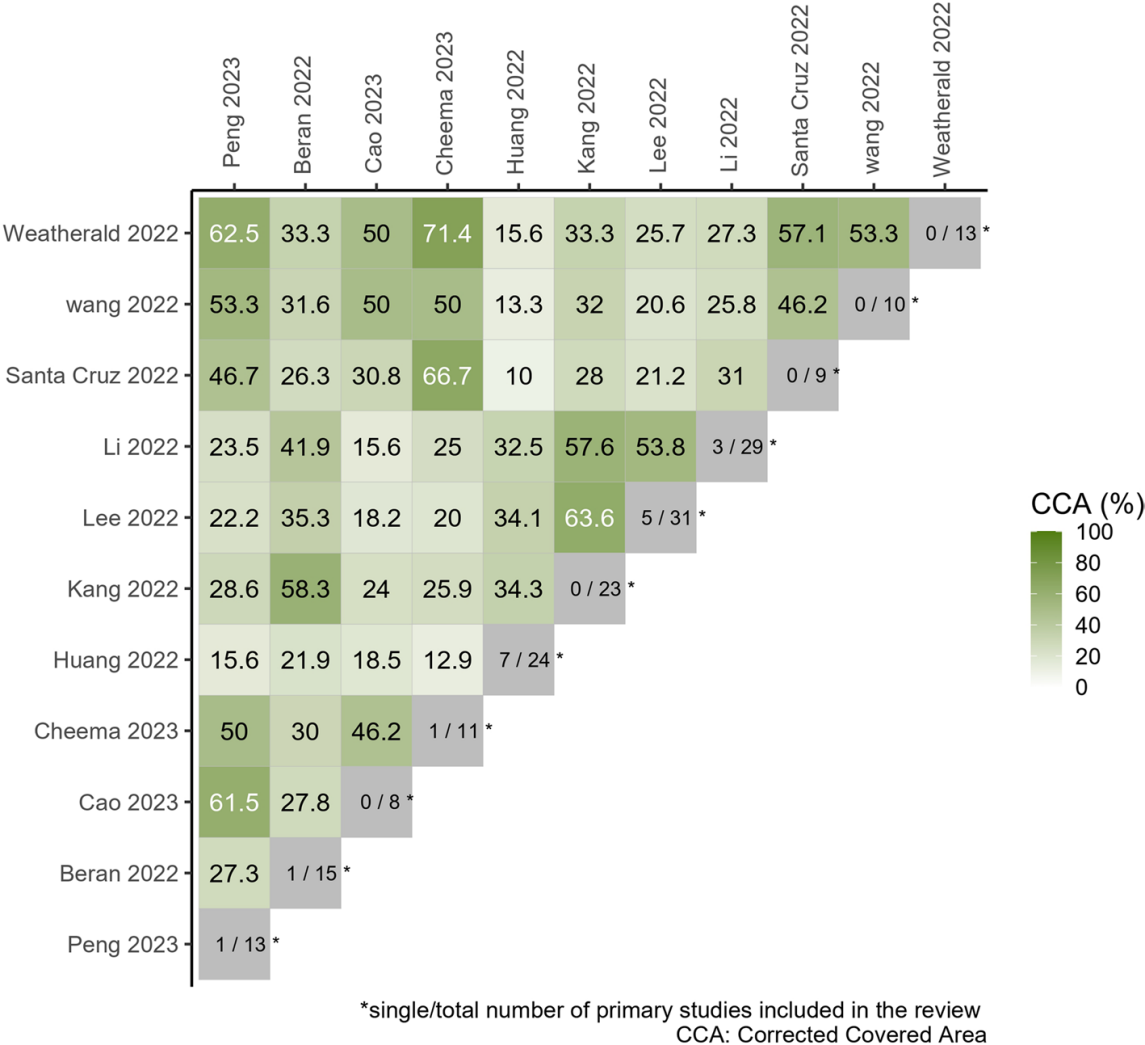
Visualization of the pairwise CCA (%) with a heatmap

This study included 11 SRs [20–30], N indicates 185, r indicates 50, and c indicates 11. The formula CCA = (185-50)/(11*50 - 50) = 27% indicated a significant level of overlap. The overlap matrix is shown in Fig. 2.

#### Methodological quality assessment

**Fig. 3:**
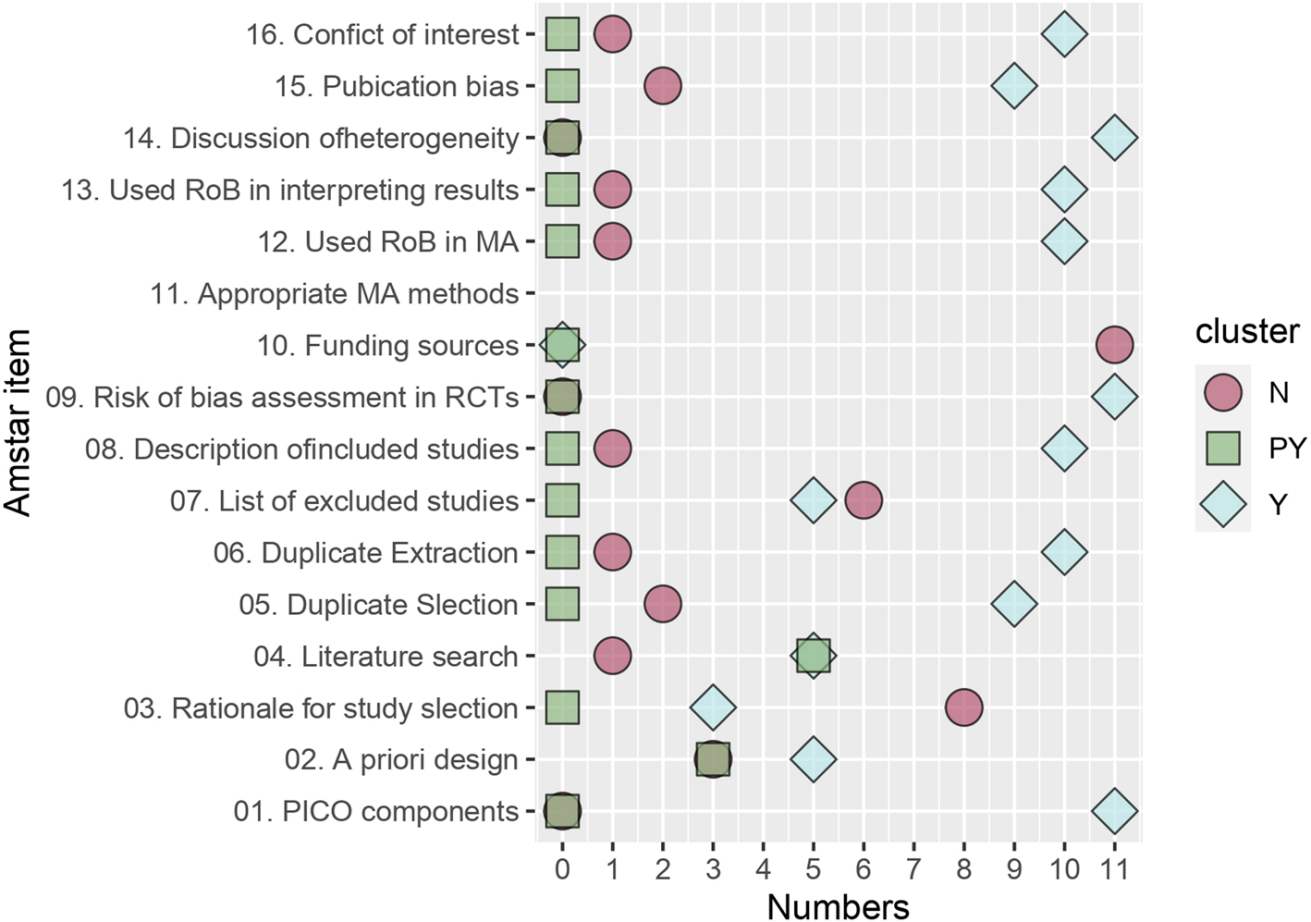
AMSTAR-2 evaluation included in systematic evaluation

The overall quality of the included studies was assessed using the AMSTAR-2 tool. Across all the covered studies, only 1 study [22] was rated as high quality, 4 studies [21, 23, 24, 26] were moderate quality, 1 study [27] was low quality and 5 studies [20, 25, 28–30] were critically low quality. Among the critical items, the following number of studies reported “Yes”: Item 2 (5/11), Item 4 (5/11), Item 7 (5/11), Item 9 (11/11), Item 11 (10/11), Item 13 (10/11), and Item 15 (7/11). For the noncritical items, none of the studies reported on Item 10, while the rest of the noncritical items were reported as “Yes” in the following numbers of studies: Item 1 (11/11), Item 3 (3/11), Item 5 (9/11), 6 (10/11), 8 (10/11), 12 (10/11), 14 (11/11), and 16 (10/11). The specific evaluation results for each item of the AMSTAR-2 in the included studies are detailed in Table 3. AMSTAR-2 evaluation included in systematic evaluation in Fig. 3.

**Table 3:** AMSTAR-2 for included SRs

Study	1	2	3	4	5	6	7	8	9	10	11	12	13	14	15	16	Overall Confidnce
Santa Cruz 2022 [20]	Y	**N**	N	**P**	Y	N	**N**	N	**Y**	N	**Y**	Y	**Y**	Y	**Y**	Y	Critically low
Cheema 2023 [21]	Y	**Y**	N	**Y**	N	Y	**Y**	Y	**Y**	N	**N**	Y	**Y**	Y	**Y**	Y	Moderate
Li 2022 [22]	Y	**Y**	Y	**Y**	Y	Y	**Y**	Y	**Y**	N	**Y**	Y	**Y**	Y	**Y**	Y	High
Huang 2022 [23]	Y	**Y**	N	**Y**	Y	Y	**Y**	Y	**Y**	N	**Y**	Y	**Y**	Y	**Y**	Y	Moderate
Kang 2022 [24]	Y	**P**	N	**Y**	Y	Y	**N**	Y	**Y**	N	**Y**	Y	**Y**	Y	**Y**	Y	Moderate
Beran 2022 [25]	Y	**N**	N	**N**	N	Y	**N**	Y	**N**	N	**N**	Y	**Y**	Y	**N**	Y	Critically low
Lee 2022 [26]	Y	**Y**	Y	**P**	Y	Y	**Y**	Y	**Y**	N	**Y**	Y	**Y**	Y	**Y**	N	Moderate
Weatherald 2022 [27]	Y	**P**	N	**Y**	Y	Y	**Y**	Y	**Y**	N	**Y**	Y	**Y**	Y	**Y**	N	Low
Wang 2023 [28]	Y	**Y**	Y	**P**	Y	Y	**N**	Y	**Y**	N	**Y**	Y	**Y**	Y	**Y**	N	Critically low
Cao 2023 [29]	Y	**P**	N	**P**	Y	Y	**N**	Y	**Y**	N	**Y**	Y	**Y**	Y	**Y**	Y	Low
Peng 2023 [30]	Y	**N**	N	**P**	Y	Y	**N**	Y	**Y**	N	**Y**	N	**N**	Y	**Y**	Y	Critically low

1: Are the research questions and inclusion criteria of the systematic review based on PICO framework? 2: Was a protocol for the systematic review developed prior to conducting the study, and if so, are the details of any revisions reported? 3: Is there an explanation provided for the selection of the study design? 4: Was a comprehensive search strategy used? 5: Does the study selection process demonstrate repeatability? 6: Does the data extraction process demonstrate repeatability? 7: Is a list of excluded studies and the reasons for exclusion provided? 8: Is detailed basic information about the included studies described? 9: Is the method for assessing bias risk in the included studies reasonable? 10: Is funding information for the included studies reported in the systematic review? 11: If meta-analysis was conducted, were appropriate statistical methods used for synthesizing the results? 12: If meta-analysis was conducted, was the impact of individual study bias risk on the meta-analysis results evaluated? 13: Was consideration given to the bias risk of individual studies when interpreting and discussing the results of the systematic review? 14: Is there a satisfactory explanation and discussion of existing heterogeneity? 15: If quantitative synthesis was performed, was the possibility of publication bias adequately investigated and discussed? 16: Are potential sources of conflicts of interest reported, including current funding resources received for the systematic review?

*Y* YES, *P *Partially Yes, *N *NO

#### Assessment of evidence quality

According to the GRADE, the quality of evidence for the outcome measures was as follows: moderate quality (14/49), low quality (17/49), and very low quality (18/49). In terms of intubation risk, 10 studies [20–24, 26–30] were rated as moderate quality, 1 study [25] was low quality, and 4 studies [22–24, 26] were very low quality. For all-cause mortality, 1 study [30] was rated as moderate quality, 9 studies [20–24, 26–29] were rated as low quality, 4 studies [22–24, 26] were rated as low quality, and 4 studies were rated as high quality. In the assessment of oxygenation, 1 study [30] was rated as low quality. Regarding ICU length of stay, 2 studies [30] were moderate quality, 1 study [21, 22] was low quality, 1 study [23] was low quality, and 4 studies [22, 23, 27, 29] were very low quality. For hospital length of stay, 3 studies [21, 22, 29] were rated as low quality, and 3 studies [22, 27, 30] were very low quality. In terms of adverse events, 4 studies [21, 24, 26, 30] were rated as moderate quality, while 4 studies [24, 26–29] were very low quality. All included primary studies were evaluated as having a high risk of bias, particularly in the areas of randomization, allocation concealment, and blinding. This was the main factor contributing to the downgrading of evidence quality. The secondary factors included imprecision (29, 58%) and inconsistency (21, 42%). The outcomes from the included SRs are summarized and presented in Table 4.

**Table 4:** Grade evaluation included in the system evaluation

Certainty assessment						№ of patients		Effect		Certainty
№ of studies	Study design	Risk of bias	Inconsistency	Indirectness	Imprecision	APP	Nonapp	Relative (95% CI)	Absolute (95% CI)	
**Santa Cruz 2022**										
**Intubation rate**	7 RCT	very serious^a^	not serious	not serious	not serious	208/717 (29.0%)	249/684 (36.4%)	**RR 0.82** (0.71 to 0.95)	**66 per 1000** (from 106 fewer to 18 fewer)	⨁⨁◯◯ Low
**Mortality**	7 RCT	very serious^a^	not serious	not serious	not serious	133/717 (18.5%)	144/684 (21.1%)	**RR 0.90** (0.72 to 1.11)	**21 fewer per 1000** (from 59 fewer to 23 more)	⨁⨁◯◯ Low
**oxygenation**	5 RCT	–				Five studies using different assessment of oxygenation (SaO2/FiO2, SaO2 and ROX index), describes a positive impact of APP in gas exchange. In turn, two studies (which assessed PaO2/FiO2 ratio) show a decrease in oxygenation. In the remaining study, oxygenation was not evaluate. Due to the different assessments of oxygenation, no metaanalysis was performed.				–
**Cheema 2023**										
**Intubation rate**	11 RCT	serious^b^	not serious	not serious	not serious	286/1218 (23.5%)	334/1167 (28.6%)	**RR 0.84** (0.74 to 0.95)	**46 fewer per 1000** (from 74 fewer to 14 fewer)	⨁⨁⨁◯ Moderate
**Mortality**	11 RCT	serious^b^	not serious	not serious	serious^c^	181/1218 (14.9%)	189/1167 (16.2%)	**RR 0.93** (0.77 to 1.11)	**11 fewer per 1000** (from 37 fewer to 18 more)	⨁⨁◯◯ Low
**ICU length of stay**	5 RCT	not serious	not serious	not serious	serious^c^	472	508	–	MD **0.88 higher** (0.96 higher to 1.12 higher)	⨁⨁⨁◯ Moderate
**Hospital length of stay**	8 RCT	serious^b^	not serious	not serious	serious^c^	857	820	–	MD **0.55 higher** (0.55 lower to 1.66 higher)	⨁⨁◯◯ Low
**Ventilator-Free days**	3 RCT	serious^b^	serious^d^	not serious	serious^c^	256	249	–	MD **3.36** (7.2 to 13.93)	⨁◯◯◯ Very low
**Adverse events**	11 RCT	not serious	not serious	not serious	serious^c^	100/1218 (8.2%)	85/1167 (7.3%)	not estimable		⨁⨁⨁◯ Moderate
**Li 2022**										
**Intubation rate**	10 RCT	not serious	not serious	not serious	not serious	216/1013 (21.3%)	255/972 (26.2%)	**RR 0.84** (0.73 to 0.97)	**42 fewer per 1000** (from 71 fewer to 8 fewer)	⨁⨁⨁◯ Moderate
**Mortality**	10 RCT	not serious	not serious	not serious	serious^c^	135/1013 (13.3%)	143/972 (14.7%)	**RR 1.00** (0.70 to 1.44)	**0 fewer per 1000** (from 44 fewer to 65 more)	⨁⨁◯◯1 Low
**ICU length of stay**	5 RCT	not serious	not serious	not serious	serious^c^	472	508	–	MD **0.08** (0.89 to 1.05)	⨁⨁⨁◯ Moderate
**Hospital length of stay**	8 RCT	not serious	serious^d^	not serious	serious^c^	875	820	–	MD **0.57** (0.35 to 1.49)	⨁⨁◯◯ Low
**Intubation rate**	18 observational studies	serious^a^	very serious^f^	not serious	not serious	254/1066 (23.8%)	626/1440 (43.5%)	**RR 0.62** (0.47 to 0.83)	**165 fewer per 1000** (from 230 fewer to 74 fewer)	⨁◯◯◯ Very low
**Mortality**	17 observational studies	serious^a^	not serious^f^	not serious	serious^c^	187/1080 (17.3%)	433/1421 (30.5%)	**RR 0.56** (0.48 to 0.65)	**134 fewer per 1000** (from 158 fewer to 107 fewer)	⨁◯◯◯ Very low
**ICU length of stay**	5 observational studies	serious^a^	very serious^f^	not serious	serious^c^	142	263	–	MD **3.38** (3.29 to 10.05)	⨁◯◯◯ Very low
**Hospital length of stay**	7 observational studies	very serious^a^	very serious^f^	not serious	serious^c^	265	371	–	MD **4.46** (12.45 to 3.53)	⨁◯◯◯ Very low
**Adverse events**	7 RCT					vascular catheters (37 patients, 2.5%) and pain or discomfort (30 patients, 2%). Other reported adverse events in the awake prone positioning groups included nausea and vomiting (17 patients, 1.2%) and skin breakdown or pressure ulcers (10 patients, 0.7%) -				
**Huang 2022**										
**Intubation rate**	10 RCT	serious^b^	not serious	not serious	not serious	274/850 (32.2%)	322/836 (38.5%)	**RR 0.84** (0.74 to 0.95)	**62 fewer per 1000** (from 100 fewer to 19 fewer)	⨁⨁⨁◯ Moderate
**Mortality**	10 RCT	serious^b^	not serious	not serious	serious^c^	175/850 (20.6%)	186/836 (22.2%)	**RR 0.92** (0.77 to 1.10)	**18 fewer per 1000** (from 51 fewer to 22 more)	⨁⨁◯◯ Low
**ICU length of stay**	5 RCT	serious^b^	very serious^f^	not serious	not serious	1066	1050	–	MD **0.58** (2.49 to 1.32)	⨁◯◯◯ Very low
**Intubation rate**	12 observational studies	very serious^a^	not serious	not serious	serious^c^	118/536 (22.0%)	485/986 (49.2%)	**OR 0.35** (0.27 to 0.46)	**239 fewer per 1000** (from 285 fewer to 184 fewer)	⨁◯◯◯ Very low
**Mortality**	10 observational studies	very serious^a^	not serious	not serious	serious^c^	51/465 (11.0%)	223/901 (24.8%)	**OR 0.34** (0.24 to 0.49)	**147 fewer per 1000** (from 174 fewer to 109 fewer)	⨁◯◯◯ Very low
**ICU length of stay**	5 observational studies	very serious^a^	not serious	not serious	serious^c^	317	325	–	MD **2.71** (4.05 to 1.37)	⨁⨁◯◯ Low
**Kang 2022**										
**Intubation rate**	7 RCT	serious^b^	not serious	not serious	not serious	344/1192 (28.9%)	417/1172 (35.6%)	**OR 0.72** (0.61 to 0.86)	**71 fewer per 1000** (from 104 fewer to 34 fewer)	⨁⨁⨁◯ Moderate
**Mortality**	7 RCT	serious^b^	not serious	not serious	serious^c^	246/1192 (20.6%)	263/1172 (22.4%)	**OR 0.89** (0.73 to 1.09)	**20 fewer per 1000** (from 50 fewer to 15 more)	⨁⨁◯◯ Low
**Adverse events**	4 RCT	serious^b^	not serious	not serious	not serious^c^	−/1026	−/1000	**OR 1.05** (0.52 to 2.11)	**0 fewer per 1000** (from 0 fewer to 0 fewer)	⨁⨁⨁◯ Moderate
**Intubation rate**	15 observational studies	serious^b^	very serious^f^	not serious	not serious	265/1166 (22.7%)	676/1616 (41.8%)	**OR 0.64** (0.48 to 0.83)	**103 fewer per 1000** (from 162 fewer to 45 fewer)	⨁◯◯◯ Very low
**Mortality**	13 observational studies	serious^b^	very serious^f^	not serious	serious^c^	192/1097 (17.5%)	433/1525 (28.4%)	**OR 0.44** (0.29 to 0.66)	**135 fewer per 1000** (from 181 fewer to 77 fewer)	⨁◯◯◯ Very low
**Adverse events**	2 observational studies	serious^b^	very serious^f^	not serious	serious^c^	−/206	−/0	**OR 6.56** (0.45 to 95.91)	**7 fewer per 1000** (from 96 fewer to 0 fewer)	⨁◯◯◯ Very low
**Beran 2022**										
**Intubation rate**	14					430/1495 (28.8%)	545/1829 (29.8%)	**RR 0.85** (0.66 to 1.08)	**45 fewer per 1000** (from 101 fewer to 24 more)	–
**Mortality**	14					263/1472 (17.9%)	455/1770 (25.7%)	**RR 0.68** (0.51 to 0.90)	**82 fewer per 1000** (from 126 fewer to 26 fewer)	–
**Hospital length of stay**								–	MD **3.09** (10.14 to 3.96)	–
**Lee 2022**										
**Need for intubation**	7 RCT	serious^b^	not serious	not serious	not serious	339/1093 (31.0%)	414/1063 (38.9%)	**RR 0.81** (0.72 to 0.90)	**74 fewer per 1000** (from 109 fewer to 39 fewer)	⨁⨁⨁◯ Moderate
**Mortality**	8 RCT	serious^b^	not serious	not serious	serious^c^	247/1219 (20.3%)	266/1185 (22.4%)	**RR 0.91** (0.78 to 1.06)	**20 fewer per 1000** (from 49 fewer to 13 more)	⨁⨁◯◯ Low
**Adverse events**	6 RCT	serious^b^	not serious	not serious	not serious	−/7011		**RR 0.97** (0.66 to 1.43)	**1 fewer per 1000** (from 1 fewer to 1 fewer)	⨁⨁⨁◯ Moderate
**Need for intubation**	18 observational studies	very serious^a^	very serious^f^	not serious	serious^c^	334/1515 (22.0%)	765/1859 (41.2%)	**RR 0.65** (0.50 to 0.85)	**144 fewer per 1000** (from 206 fewer to 62 fewer)	⨁◯◯◯ Very low
**Mortality**	18 observational studies	very serious^a^	serious^d^	not serious	serious^c^	275/1496 (18.4%)	586/1865 (31.4%)	**RR 0.56** (0.45 to 0.70)	**138 fewer per 1000** (from 173 fewer to 94 fewer)	⨁◯◯◯ Very low
**Adverse events**	6 observational studies	very serious^a^	serious^d^	not serious	serious^f^	–				⨁◯◯◯ Very low
**Weatherald 2022**										
**Intubation rate**	13 RCT	serious^b^	not serious	not serious	not serious	293/1211 (24.2%)	343/1125 (30.5%)	**RR 0.83** (0.73 to 0.94)	**52 fewer per 1000** (from 82 fewer to 18 fewer)	⨁⨁⨁◯ Moderate
**Mortality**	13 RCT	serious^b^	not serious	not serious	serious^c^	189/1199 (15.8%)	196/1140 (17.2%)	**RR 0.90** (0.76 to 1.07)	**17 fewer per 1000** (from 41 fewer to 12 more)	⨁⨁◯◯ Low
**ICU length of stay**	7 RCT	serious^b^	serious^f^	not serious	serious^c^	2290	2190	–	MD **1.78** (3.81 to 0.24)	⨁◯◯◯ Very low
**Hospital length of stay**	7 RCT	serious^b^	serious^f^	not serious	serious^c^	2290	2190	–	MD **0.02** (0.93 to 0.98)	⨁◯◯◯ Very low
**ventilator free days**	4 RCT	serious^b^	not serious	not serious	serious^c^	2085	1977	–	MD **0.52** (0.19 to 1.24)	⨁⨁◯◯ Low
**oxygenation**	16					Significant heterogeneity in the reported oxygenation indices and time of outcome assessment precluded pooling of data.				–
**Adverse events**	12					vascular catheters (37 patients, 2.5%) and pain or discomfort (30 patients, 2%). Other reported adverse events in the awake prone positioning groups included nausea and vomiting (17 patients, 1.2%) and skin breakdown or pressure ulcers (10 patients, 0.7%)				–
**Wang 2022**										
**intubation**	9 RCT	not serious	serious^b^	not serious	not serious	281/1172 (24.0%)	329/1122 (29.3%)	**RR 0.84** (0.74 to 0.95)	**47 fewer per 1000** (from 76 fewer to 15 fewer)	⨁⨁⨁◯ Moderate
**mortality**	9 RCT	not serious	serious^b^	not serious	serious^c^	177/1172 (15.1%)	187/1122 (16.7%)	**RR 0.93** (0.77 to 1.11)	**12 fewer per 1000** (from 38 fewer to 18 more)	⨁⨁◯◯ Low
**Adverse events.**	7 RCT	not serious	serious	not serious	very serious	108/1147 (9.4%)	85/1090 (7.8%)	**RR 1.16** (0.48 to 2.76)	**12 more per 1000** (from 41 fewer to 137 more)	⨁◯◯◯ Very low
**Cao 2023**										
**intubation rate**	8 RCT	serious^b^	not serious	not serious	not serious	347/1351 (25.7%)	423/1306 (32.4%)	**OR 0.72** (0.60 to 0.86)	**67 fewer per 1000** (from 101 fewer to 32 fewer)	⨁⨁⨁◯ Moderate
**mortality**	8 RCT	serious^b^	serious^c^	not serious	not serious	249/1351 (18.4%)	268/1306 (20.5%)	**OR 0.88** (0.72 to 1.08)	**20 fewer per 1000** (from 48 fewer to 13 more)	⨁⨁◯◯ Low
**ICU length of stay**	2 RCT	serious^b^	very serious^g^	not serious	not serious	45	45	–	MD **1.14** (0.45 to 2.72)	⨁◯◯◯ Very low
**Hospital length of stay**	2 RCT	serious^b^	serious^c^	not serious	not serious	579	572	–	MD **0.11** (1.02 to 1.23)	⨁⨁◯◯ Low
**Adverse events.**	6 RCT	not serious	serious	not serious	very serious	1306/143	1261/135	**OR 1.02** (0.79, 1.31)		⨁◯◯◯ Very low
**Peng 2023**										
**intubation rate**	13 RCT	serious^b^	not serious	not serious	not serious	431/1674 (25.7%)	518/1589 (32.6%)	**OR 0.72** (0.61 to 0.84)	**68 fewer per 1000** (from 98 fewer to 37 fewer)	⨁⨁⨁◯ Moderate
**mortality**	10 RCT	serious^b^	not serious	not serious	not serious	306/1647	325/1576	**RR 0.88** (0.74 to 1.06)	**-- per 1000 patient(s) per years** (from -- to --)	⨁⨁⨁◯ Moderate
**Hospital length of stay**	8 RCT	serious^b^	not serious	not serious	very serious^f^	2267		–	MD **0.36** (1.39 to 0.66)	⨁◯◯◯ Very low
**PaO2/FiO2**	5 RCT	serious^b^	serious^f^	not serious	not serious	584	561	–	MD **29.76** (11.39 to 48.13)	⨁⨁◯◯ Low
**Adverse events.**	9 RCT	serious^b^	serious^f^	not serious	not serious	1568/165	1499/135	**OR 1.21** (0.58, 2.54)		⨁⨁⨁◯ Moderate

*CI* confidence interval, *MD* mean difference, *OR* odds ratio, *RR* risk ratio

Explanations

a. Most information was from studies at high risk of bias, with large flaws in randomization methods, allocation concealment, or blinding or without risk of bias assessment;

b. There are certain shortcomings in randomization methods, allocation hiding or blinding methods

c. Insufficient sample size or wide confidence interval

d. Greater heterogeneity included in the study

e. Left-right asymmetry in funnel diagrams

f. Studies with large heterogeneity included in the study and without heterogeneity analysis

g. Insufficient sample size and wide confidence intervals

### Effects of interventions

#### Intubation rate

A total of 11 SRs [20–30] reported intubation risk in COVID-19 patients. Meta-analyses demonstrated that regardless of study design (RCTs or observational studies), APP significantly reduced intubation risk (*P* < 0.05). However, Santa Cruz [20] conducted a sensitivity analysis and found that this benefit was not sustained after excluding the study with the highest weight. Furthermore, subgroup analyses were performed in eight studies [21, 22, 24, 26–30] to investigate factors such as the modality of respiratory support (conventional oxygen therapy versus higher levels of respiratory support), enrollment location (ICU versus non-ICU), median duration of APP use per day, and baseline SpO_2_/FiO_2_ ratio. The subgroup analyses revealed a significant reduction in intubation risk among patients receiving higher levels of respiratory support, those enrolled in the ICU, those who underwent prone positioning for more than 5 or 8 hours, and those with baseline SpO_2_/FiO_2_ ≥ 235 mmHg. However, the nonsignificant subgroup difference *p* values [21, 22, 24, 26–30] and the high overlap of confidence intervals [25, 26] among the included studies confirm that there is no significant interaction between the mentioned factors and the intubation rate.

#### Mortality

Eleven SRs [20–30] reported all-cause mortality. Among these, 7 studies conducted meta-analyses using only RCTs and found no statistically significant difference between groups (*P* > 0.05). Four studies explored the influence of APP on the risk of mortality in COVID-19 patients using observational studies. They found a significant reduction in mortality with APP (*P* > 0.05), but significant heterogeneity was observed among the studies. Beran et al. [25] conducted a pooled analysis combining five RCTs and nine observational studies and found a statistically significant difference between the groups (*P* < 0.05, *I*^*2*^ = 52%). Subgroup analyses [21, 22, 24, 26, 28, 29] examining various factors (type of respiratory support, enrollment location, APP daily median duration, baseline SpO_2_/FiO_2_ ratio) did not reveal any significant interactions with mortality based on the nonsignificant subgroup difference *p* values [21, 22, 24, 26–30] and the high overlap of confidence intervals [25, 26].

#### Oxygenation

Three studies [20, 23, 30] reported on the improvement in oxygenation. Peng et al [20] demonstrated that APP significantly improved the PaO_2_/FiO_2_ ratio (MD 29.76[11.39, 48.13], *P* < 0.001, *I*^*2*^ = 96%), and Santa Cruz [20] was unable to draw conclusions regarding improvements in oxygenation due to the use of different criteria for assessing oxygenation across the five RCTs included in their study. One study [23] did not perform data pooling for improvements in oxygenation due to high heterogeneity observed in the oxygenation index.

#### ICU length of stay

Five studies [21, 22, 26, 27, 29] examined the length of ICU stay. The MAs did not reveal any statistically significant difference in the length of ICU stay between the APP and control groups. The subgroup analyses [22], investigating the type of respiratory support and enrollment location, both showed overlapping confidence intervals within each subgroup, indicating that there is no significant interaction between these factors and the ICU stay duration.

#### Hospital length of stay

Among the included SRs, seven studies [21, 22, 25–27, 29, 30] reported on the length of hospital stay. The MAs of both RCTs and observational studies showed no statistically significant difference in the length of hospital stay between the APP and control groups (*P* > 0.05). The subgroup analyses [22] investigating the type of respiratory support and enrollment location showed overlapping confidence intervals, indicating that based on the existing evidence, these two factors are likely not significantly interacting with the duration of hospitalization.

#### Ventilator-free days

Three studies [21, 26, 27] reported on ventilator-free days as an outcome measure. The results revealed no statistically significant difference between the APP and control groups in terms of ventilator-free days (*P* > 0.05).

#### Adverse events

Eight studies [21, 22, 24, 26–30] reported adverse events. A pooled analysis of six studies [21, 24, 26, 28–30] revealed that there was no significant difference in the incidence of adverse events between the APP group and control group (*P* > 0.05). Two studies [19, 24] reported specific adverse events, such as pain or discomfort, accidental dislodgement of the vascular catheter, nausea and vomiting, skin damage or pressure ulcers, abdominal distension, and general discomfort. The incidence of adverse events was comparable between the two groups.

## Discussion

This overview encompasses 11 SRs to assess and summarize the evidence on the safety and efficacy of APP for treating COVID-19-related acute hypoxemic respiratory failure. According to AMSTAR 2, only 1 SR [22] was rated as high quality. The main reasons are related to suboptimal practices in key items, including: 1. Partially registered study protocols may lead to selective reporting bias. 2. Insufficient justification for the selection of study types, such as some studies included semirandomized controlled trials or other types without adequate rationale. 3. Incomplete literature searches were conducted, as many studies failed to search professional registration platforms and overlooked the retrieval of gray literature. 4. The absence of disclosure regarding funding sources or conflicts of interest potentially influences the impartiality of the SRs’ results. Improved methodological rigor is needed in SRs, which should begin with a well-designed protocol and implement rigorous control of bias risks throughout the process. Tools such as AMSTAR 2 can be used to standardize the review process. The reliability of systematic review findings depends on the entire production process. Improving methodological and reporting quality will enhance the translational potential of interventional reviews, making them more persuasive.

The focal point for evaluating clinical efficacy lies in outcome measures. Based on the SRs included in this study, consistent results demonstrate a significant improvement in intubation rates among patients with APP despite varying criteria and indications. However, sensitivity analysis suggests that certain studies may influence these findings. Results from three SRs indicate that APP demonstrates advantages in improving oxygenation (PaO_2_/FiO_2_, SpO_2_, PaO_2_) in patients who are spontaneously breathing or undergoing NIV/ HFNC therapy. However, it was observed that not all patients were able to maintain these improvements in oxygenation after reverting to the supine position. This variability in response may be attributed to several factors. Firstly, the hypoxemia associated with COVID-19 is multifactorial in nature, and different respiratory support modalities operate through varied mechanisms [31, 32]. This leads to differential responses to APP in patients with ARDS related to COVID-19. Secondly, the SRs in our research show a lack of uniformity in critical aspects such as the timing of initiation of prone positioning, the severity of hypoxemia, the underlying causes, types of infiltration, and other relevant data. Moreover, there is a lack of RCTs specifically exploring the impact of APP on oxygenation improvement in patients with COVID-19-related ARDS. In terms of mortality, the conclusions from SRs of different study types are often contradictory. Positive results are often driven from SRs of observational studies and RCT-based MAs showing no reduction in mortality with APP, which may be attributed to several factors. First, basic characteristics of patients such as age, illness severity, and individual tolerance. Additionally, a lack of standardized protocols, timing of APP initiation, inadequate actual duration of APP, limited follow-up periods, and small sample sizes may collectively contribute to insufficient statistical power in detecting differences in mortality. An increased duration of APP was found to be associated with a lower risk of intubation. However, it’s crucial to note that this evidence is solely supported by the results of subgroup analysis in MAs and should be interpreted with due caution. Previous studies [5, 33] have shown that early application of at least 12 hours of prone positioning can improve survival rates in patients with moderate to severe ARDS. Current available data also indicate that COVID-19 patients who can tolerate longer proning sessions, specifically ≥6 to 8 hours, may experience benefits from prone positioning [34, 35]. However, in majority of SRs included in this study, the duration of APP ranged from 1 to 2 hours/day to 8 to 10 hours/day. APP time completely depends on patient comfort and tolerance, patient compliance and tolerance in the conscious state often prove inadequate, resulting in actual daily APP duration falling significantly short of expectations, which may not suffice to attain survival benefits. Therefore, various techniques, such as rotational and lateral positioning, frequent proning, patient tracking records, or mild sedation, have been investigated to enhance patient compliance and tolerance during APP. Further validation is required to establish the dose–response relationship between the duration of APP and its effectiveness.

In terms of adverse events, the incidence rate was comparable, and no serious adverse events were reported, suggesting that the utilization of APP in COVID-19 patients under close medical supervision may represent a viable and safe option. Although the use of APP may temporarily improve oxygenation in some patients, this could potentially delay intubation and invasive ventilation and increase the risk of self-inflicted lung injury and mortality [36]. Therefore, individual patient characteristics, disease severity, and institutional resources must be considered when deciding on prone positioning. Close monitoring of patients’ response to prone positioning and oxygenation is essential, with timely intubation if necessary to prevent delays and potential harm. Future studies should prioritize safety, proactively use tools such as foam cushions and gel rings, provide continuous education to healthcare providers on prone positioning, and improve patient compliance to reduce complications.

The GRADE evaluation of the quality of evidence for outcome measures in this study also indicates a lack of high-quality evidence. The main reason for downgrading the outcome measures is the low methodological quality of the included primary studies, with deficiencies observed in randomization, allocation concealment, and blinding. Other reasons for downgrading the evidence to low quality include ① the presence of publication bias without any bias source analysis; ② the small sample size leading to wide confidence intervals for the pooled effect estimates, indicating imprecision; and high heterogeneity among the included studies without discussion and analysis of the sources of heterogeneity, resulting in downgrading for inconsistency.

## Limitations

Despite conducting comprehensive research and evidence synthesis, our review has several limitations. We only included SRs published in Chinese and English languages. This approach may lead to insufficient coverage of relevant studies and incomplete evaluation due to publication and regional biases. Although we performed cross-checking in the methodology and evidence quality assessment, some evaluation items might still be influenced by subjective factors of the evaluators, potentially leading to biased results. The presence of overlap of primary RCTs among the included reviews may restrict the interpretation of our results. The SRs included in our study exhibited significant variation in terms of study design, patient populations, interventions, and outcome measures. The uncertainty in the original data may translate to additional uncertainty in the secondary studies, warranting cautious interpretation of the reported results.

## Conclusion

Based on the available SRs, APP may have potential benefits in COVID-related acute hypoxaemic respiratory failure, although the current evidence is limited and of low quality. Clinicians should carefully weigh the potential benefits and risks and individualize the treatment approach for each patient. Further research is needed to address the existing limitations and provide more robust evidence on the effectiveness and safety of APP in COVID-related acute hypoxaemic respiratory failure.

### Supplementary Information

**Additional file 1.**

## Data Availability

All data generated or analysed during this study are included in this published article and its supplementary information files.

## Abbreviations

RCTs: Randomized controlled trials
ICU: Intensive care unit
OR: Odds ratio
MD: Mean difference
CI: Confidence interval
APP: Awake prone positioning
SRs: Systematic reviews
CCA: Corrected covered area
EDs: Emergency departments
